# Energy transition awareness: Can it guide local transition planning on islands?

**DOI:** 10.1016/j.heliyon.2023.e19960

**Published:** 2023-09-09

**Authors:** Andrew Barney, Heracles Polatidis, Stergios Vakalis, Dominique Grondin, Michel Benne, Fausto Sainz Salces, Dias Haralambopoulos

**Affiliations:** a Uppsala University, Dept. of Earth Sciences, Cramérgatan 3, 621 57, Visby, Sweden; b University of the Aegean, Dept. of Environment, University Hill, 81100, Mytilene, Lesvos, Greece; c ENERGY Lab, University of La Réunion, 15 Avenue René Cassin CS 92003, CEDEX 9, 97744, Saint-Denis, France; d International University of La Rioja, College of Engineering and Technology (ESIT), Avenida de la Paz, 137. 26006, Logroño, Spain

**Keywords:** Decarbonisation, Islands, Energy transition, Energy awareness, Consumer engagement

## Abstract

The consequences of climate change and reduced energy security are becoming increasingly apparent, especially on islands. At the same time, the energy transition is quickly spreading and its value to society becoming clearer. Two main obstacles to this transition, rigid policy and lack of local understanding, are particularly troubling on islands, where national policies often aren't flexible enough to consider local particularities and residents are exposed to different energy realities from those on the mainland. Using exploratory interviews and a survey on four islands, this article considers island residents' awareness of energy transition concepts and presents how it interacts with, and is potentially influenced by, relevant energy policies at the national level. The paper presents the comparative results for the geographically, demographically and climatologically diverse islands of Gotland (Sweden), Lesvos (Greece), La Réunion (France) and Mallorca (Spain) to focus on European island energy transitions. Differences were noted between the islands' residents with regards to awareness of and willingness to use specific energy transition tools or to join activities like energy communities. Additionally, differences were noted between the islands for what was the most important reason to consider when using demand response, though ‘Ease of use’ was important across all. The potential reasons for differences among the islands are discussed and suggestions to increase consumer engagement with energy transition activities on islands are given. Overall, the results show that while awareness of energy concepts isn't greater on these European islands, interest in prospective transition actions was high and provide an opportunity for planners to capitalize on. However, if there are potential policy obstacles, these higher levels of interest cannot ensure higher levels of willingness to engage. Taken together, these two findings indicate the potential for an acceleration of transition activity on these islands, and potentially beyond, should engagement with island residents be increased along with review, and amendment of policies impeding it.

## Nomenclature

G##: Gotland respondent and number
L##: Lesvos respondent and number
M##: Mallorca respondent and number
R##: La Réunion respondent and number
TOU: Time of Use

## 1 Introduction

Island residents find themselves vulnerable to climate change's threats to their local environments and livelihoods and may have greater difficulty than mainlanders in developing appropriate reactions to these threats due to their remoteness and, often, more limited resources. At the same time, energy security stands out as a key issue for islands and constitutes a significant challenge for energy and environmental planners [1].

Research on energy transition isn't new and is only becoming more relevant as climate change's impacts increase. This is especially the case for islands and island communities that have needs and requirements not always mirrored in national policies. Research on energy transition for islands ranges from integrating renewable energy and energy storage technologies into existing energy structures [2,3], attempting to achieve island energy independence [4], suggesting ways the transition can be better supported on islands [5], or developing decision-aid methods to assist in the planning for the energy transition [6].

Developing effective policy and engagement measures for the energy transition on islands is difficult without first understanding the islands' inhabitant's starting points for energy awareness and actual willingness to participate in energy transition activities. This paper's objectives are to determine both of these conditions on islands within the EU while also considering the local circumstances to evaluate how these circumstances, in turn, interact with the attitudes of the island inhabitants.

### 1.1 Island energy transition planning

To help address island energy security concerns, the European Union's Horizon 2020 research and innovation program has established a number of projects to develop solutions, among these is the REACT (Renewable Energy for Self-Sustainable Islands) project. The REACT project seeks to help islands achieve energy independence through increased local renewable energy generation [[6], [7], [8], [9]], creation of a demand response platform [10] and engagement with users in the local communities [7,11].

The REACT project islands’ under consideration are Gotland (Sweden), Lesvos (Greece), Mallorca (Spain) and La Réunion (France). The differences in nations, populations, economic structures, energy systems, and climates of these four islands provide a wide range of valuable information about island approaches and understandings of energy transition. Further, these differences can offer insights into a broader group of islands than an examination of a narrower, homogenous selection. Table 1 below summarizes some details of the islands included.

**Table 1:** Island detail summary for 2021 [8,[12], [13], [14], [15]].

Island	Location	Population	Area (km^2^)	Installed Renewable Energy Source Capacity (MW)	% Share of Renewable Energy Sources of Total Electricity Generated	Interconnected
Gotland	Baltic Sea	60,000	3,200	187	**∼**50%	Yes
Lesvos	Aegean Sea	84,000	1,600	21	**∼**19%	No
Mallorca	Balearic Sea	900,000	3,600	155	**∼**7%	Yes
La Réunion	Indian Ocean	860,000	2,500	630	**∼**28%	No

**Fig. 1:**
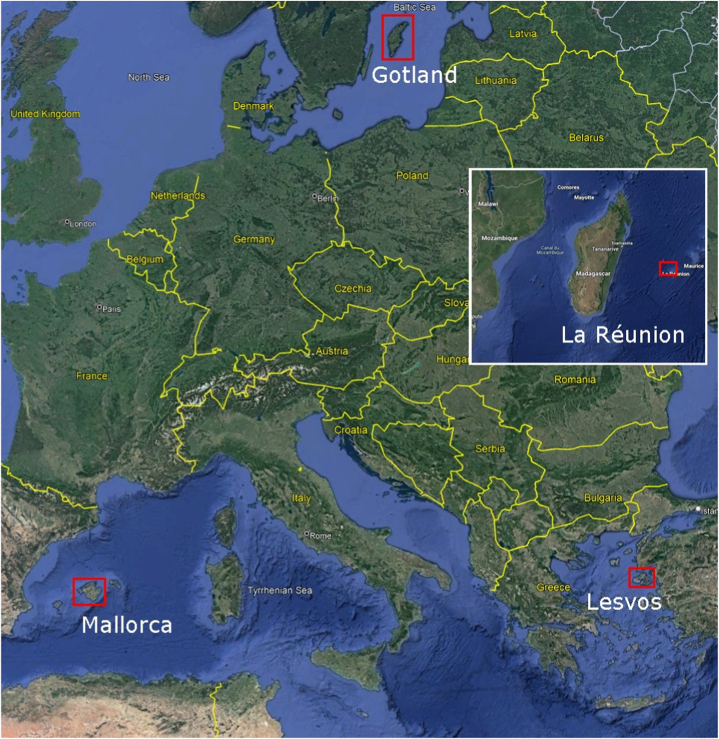
REACT islands considered in this work, La Réunion inserted [16].

The map below (Fig. 1) shows the locations of the islands in relation to each other, excepting La Réunion that has been inserted due to its distance from the European mainland.

Gotland is involved in a number of governmentally funded initiatives investigating the energy transition potential of the island [12,17] as well as specific steps for transition like investigation of storage alternatives [18] and smart grid technologies [19]. A good deal of consideration has also been given to the potential methods to increase La Réunion's renewable energy sources (RES) utilization [[20], [21], [22]], including technical paths to achieving energy autonomy [23,24]. Research for the island of Mallorca has largely focused around improving the sustainability of its tourism industry [[25], [26], [27]]. Further consideration has also been placed on decarbonizing its energy production together with the other Balearic Islands [28] and policies to help with their climate mitigation and adaptation [29]. Energy transition research on Lesvos has considered how to design the island's electricity system [30] as well as how to increase RES participation in local energy production, such wind powered pumped hydro storage [31], generation scheduling strategies [32] and interconnection with the mainland grid [33].

### 1.2 Residential consumer perceptions and acceptance of energy transition

As has been made clear, accounting for the social aspects of energy is important [34], and these aspects are undiminished, and are potentially amplified, on islands. Researching, understanding and including these local social aspects and attitudes contribute to improved acceptance, to the benefit of the development and implementation of energy transition measures [[35], [36], [37], [38]].

#### 1.2.1 Engagement and acceptance on islands

Perception and understanding of energy transition vary, depending on the location and tools being used to make that transition, but engagement has been repeatedly found to be valuable to the acceptance of energy transition projects and its surrounding planning on islands. The attitudes of residents on three islands were evaluated to determine how prepared residents were to engage with demand response and related technologies, with mixed findings [11]. While residents demonstrated high levels of willingness to change consumption behaviors and to use technologies that helped their economic condition and the environment, they are unfamiliar with the specific technologies and related concepts. This lack of awareness was determined to potentially negatively impact the resident's acceptance and underlined the importance of engaging them to increase awareness and knowledge. The energy literacy and attitudes of different households on the island of Cyprus were studied in order to provide guidance in the designing of the island's energy saving policies as well as encouraging the spread of information on these policies to residents [39]. Multiple islands across the Aegean Sea were surveyed to determine their residents' general openness to various types of energy transition technologies and systems [38]. This survey found a wide range of attitudes, generally positive, towards RES technologies but less than a quarter of those surveyed were open to the full implementation of the proposed transition solution. An understanding of local consumers' views and knowledge of existing and new RES technologies, including their perceptions of the technologies' impacts, was sought on the Greek island of Andros [35]. It was found that residents were generally positive to RES production technologies, in particular those they were most familiar with.

On Portugal's Culatra Island, islanders were engaged to participate in the planning and implementation of the island's energy transition [40]. This engagement was found to increase the speed of decision making while also improving the levels of both awareness and acceptance of the energy transition initiatives. Residents of the Irish island of Inis Oírr were involved in developing energy transition scenarios for their island, where residents themselves presented their understandings, concerns and needs to guide scenario development [41]. The residents on the island expressed the most support for the scenarios which best incorporated their input. In a survey of the Attica region of Greece, which includes a number of islands, it was found that the most important measure influencing attitudes towards RES projects was an open and continuous communication with the locals about the project's development at all project stages [42].

#### 1.2.2 Energy literacy

The impact energy literacy, or how much energy-linked knowledge someone has and how they act on it, has on motivations to engage in energy transitions has also been studied from a number of directions. Research on energy literacy among residential consumers has produced mixed findings depending on what type of literacy is being evaluated. Research on consumer's knowledge of household devices' energy usage provided mixed and dubious results [43], while the literacy of which actions a residential consumer can take to reduce energy consumption have been found to be low, in the latter case only around 50% of those involved were found to have had this knowledge [44].

A lack of consumer technological awareness or familiarity has been often identified as a barrier or impediment to the uptake of the technologies at the core of the energy transition [11,[45], [46], [47]] while [48] specially suggest policy measures to increase this awareness. In a specific case related to the investigation of the motivations, enablers and barriers for consumer engagement with residential demand response, it was further found that low awareness of the idea of demand response can result in lower levels of trust in those implementing or supporting it and can increase reluctance in using the demand response services [49].

#### 1.2.3 Technology acceptance model

The Technology Acceptance Model (TAM) is a popular way used to assess the acceptance of new technologies and to help determine consumers' perceived attitude towards them based on the technology's usefulness and its ease of use. Positive attitudes based on these two combined criteria are assumed to indicate that a person will use the technology [50]. The TAM, or one of its variations, have been used in a number of studies, either on its own or combined with another model, to evaluate consumer willingness to accept a number of different technologies including renewable energy production technologies [48,51] smart meters [52], Smart Grid technologies [53] and to measure willingness to engage with low energy communities [54], among many other applications.

#### 1.2.4 Demand response

Demand response is a piece of the greater concept of demand side management (DSM) which itself can be implemented as a component of smart grids (SG), or electrical grids that deliver electricity to consumers in a smart, controlled way. SGs rely on consumers to flexibly modify their consumption behaviors based on price signals, and demand response is the consumer's change in their consumption. Ultimately, demand response leads to economic benefits for both the consumers and utilities while also allowing greater integration of RES as a result of the increased flexibility in the energy system. There are a number of tools and methods available to assist with and encourage demand response [55,56].

### 1.3 Article contribution and structure

This paper seeks to provide a clearer understanding of the attitudes, awareness and behaviors of island inhabitants around their usage of energy and their willingness to engage with energy transition activities and tools than currently exists. By doing so, this paper generates new understandings of islanders' complex relationships with energy use and the energy transition. This increased understanding of island inhabitant's positions is expected to provide initial guidance to energy and policy planners on islands across the EU in which areas they can best engage their residents [39,40]. Further, it will allow for a better determination of which actions being already undertaken on other islands would be best to consider on the islands included in this work.

The paper's objectives are reached using the responses from interviews and a survey from four islands in the EU, Gotland, Lesvos, Mallorca and La Réunion. These responses were analyzed for each island in relation to its own conditions before being then compared to identify potential opportunities and barriers for island energy transition planning.

The structure of the paper is as follows: Chapter 2 describes the methodology in developing the interview questions and survey and then the implementation of the interviews and survey along with their relevant details. Chapter 3 presents and compares the results of the interviews and survey for the four islands. In Chapter 4, a discussion of the results is given together with observations, explanations and remarks on the existing obstacles to energy transition. Chapter 5 concludes with the paper's findings.

## 2 Methodology, design and implementation

A semi-structured interview format was selected to both inform participants about the goals of the REACT project as well as to gather information on the participant's knowledge of their own energy usage and their familiarity of key energy transition concepts. It should be noted that no attempt was made to verify the accuracy of the respondent's self-reported levels of knowledge. The respondent's willingness to join a local energy community and to employ a project related energy transition technology, demand response software, was assessed, as were their reasons for doing so. In addition to the REACT project's ethics committee, three additional ethics committees from the interviewing organizations were consulted to confirm appropriate actions in line with local and EU directives would being undertaken with regards to the both the gathering and handling of all collected data. Consent was obtained from each respondent before the interview began. The base English template for the interviews can be found in Appendix A.

The template for the interview was developed for all islands and contained 25 questions. These base questions are broken down into categories regarding respondent demographics (6), respondent energy literacy (6), energy transition concept/activity awareness (5), willingness to participate in energy transition activities (3) along with open-ended questions related to energy transition on their island and their concerns (5). The respondents on all islands were asked the questions in the same order. The template was translated into the local language for each island and some questions were slightly amended or removed, as they did not all apply for all islands. Some guidance was given in the template to the person conducting the interview on when to ask follow up questions. Additionally, respondents could provide comments throughout the interview, which were recorded.

Some questions required knowledge of an underlying energy transition concept, to be answered in a meaningful manner. If the respondent, in a question posed earlier in the interview, stated they had no knowledge of the concept, the interviewer was directed to give a pre-determined definition of the concept. The interviewer was also directed to answer any follow-up questions that arose from these definitions before asking the follow-up related question. In some instances, the respondent's answer on the open-ended question revealed that they had potentially overstated their knowledge on a topic. In these cases, the interviewer did not correct the respondent nor update the interviewee's self-assessed level of knowledge on a concept, but did note the potential discrepancy.

The interviews took place in 2021 and they were conducted in a variety of formats for three of the islands, including in-person, telephonic and digital meetings. Individuals on the islands were purposively selected [57] for their interviews on the basis that they were residents on the island, were over the age of 18, owned or rented a home and paid their electricity bill directly to the provider. The interviews were conducted via convenience sampling with snowballing [58,59]. Each interview tended to last between one and one and a half hour. For the island of La Réunion, an online survey was used instead of interviews and links to the survey were disseminated through social media.

Data collected from the interviews and the survey allow for an initial assessment of local energy transition awareness on these islands, as well as provide an early stage rough estimate of willingness to accept specific technological interventions [60] and participate in potential local transition activities [38].

In total, 20 interviews were conducted on the island of Gotland, 21 were conducted on the island of Lesvos and 23 on the island of Mallorca. A total of 89 questionnaires were begun for the online survey conducted on La Réunion of which 49 were completed in full.

**Fig. 2:**
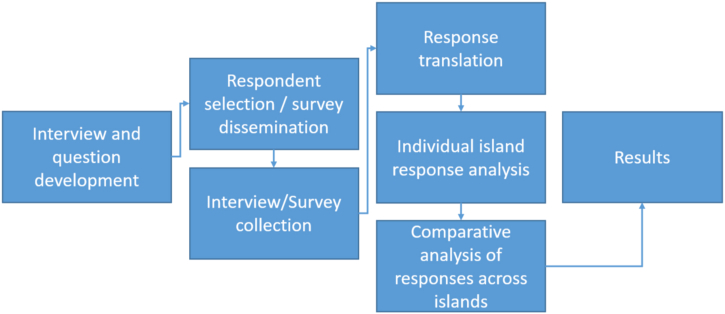
Interview and survey process and analysis.

A full outline of the methodology used in this paper is shown in Fig. 2.

The interviews were collected in Microsoft Word before being translated to English and transferred to Microsoft Excel to conduct a comparative frequency analysis of the responses across the islands to determine the differences between the different islands' respondents. Conceptual and relational content analysis was also used for the open-ended questions to establish and organize the meanings behind repeating concepts both on single islands and across the islands as well as to analyze respondents’ expressed attitudes so overall conclusions could be drawn from the responses [61,62].

A limitation of this work is that, as the respondents for the interviews and survey on some of the islands are not closely representative of the islands' populations, it is difficult to determine if their responses are generalizable to the rest of their island's population [61]. Further, given the small number of participants compared to the populations of the islands statistical interpretation was not deemed reasonable. Additionally, both the interviews and survey could suffer from a self-selection bias, only those interested in the topic likely took the time to participate. Nonetheless, as these interviews and survey were exploratory in nature the responses received are still considered to provide valuable information for planning purposes.

## 3 Results

The results from the interviews and survey on the four islands are presented here with a focus on the areas of greatest similarity and difference. This chapter presents the findings from each category of question types, as described in Chapter 2.

### 3.1 Respondent energy literacy

Several points of interest emerge when comparing the energy literacy of the respondents' across the islands. On all four islands, the majority of respondents stated that they knew the cost of the electricity they used each month as well as if it had changed over time. The majorities varied however, on Gotland and La Réunion only slightly more than half of the respondents stated they knew while on Lesvos nearly all respondents of stated they knew. This difference in knowledge between the islands’ respondents disappeared when asked how many kWh they had used each month, with only a third of the respondents on each islands stating they knew.

Awareness that electricity had different costs at different times of day, time of use (TOU) pricing, was high across all islands, ranging from just over half of respondents on La Réunion to nearly all on Mallorca. Despite this awareness, only a few respondents used such a tariff on Gotland and Mallorca. The general attitude towards TOU pricing by those that didn't use it on Gotland and Mallorca is typified by responses such as,

“Yes, I know about (TOU pricing) but I don’t bother with it.” (M02)

“Yes (I know about), but no (I don’t use TOU pricing). The prices are rather stable and I am satisfied with the tariff I have.“(G04)

In addition, in the case of those with a family, a respondent from Sweden explains,

“We don't use (TOU pricing) though and instead have a fixed price. It became very difficult to match our consumption with the price with a family and kids.” (G01)

**Fig. 3:**
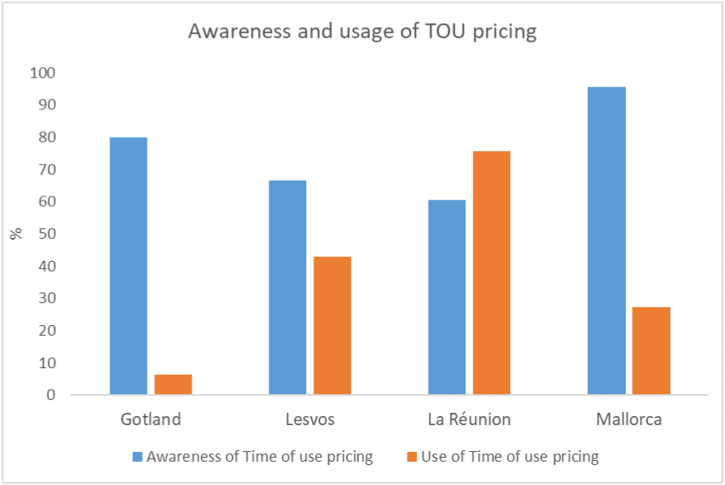
Awareness of Time of Use (TOU) pricing and percentage of island residents that know about it and use it, by island.

At the same time, despite having the lowest level of awareness, La Réunion's respondents were much more likely to use TOU tariffs with nearly three quarters of those that were aware stating they took advantage of it. The differences between awareness and use of TOU pricing by respondents on each island are shown in Fig. 3.

The number of respondents across all islands that stated they considered an appliance's energy efficiency was uniformly high. Conversely, the number owning renewable electricity generating equipment and electrical vehicles was low on all islands, though a large number across all islands stated they would consider purchasing an electric car.

“Yes, I have been looking at a hybrid.“(M09)

“Yes. I'd buy an electric car if I had enough money. Maybe in the next five years when they become cheaper and better.“(G06)

**Fig. 4:**
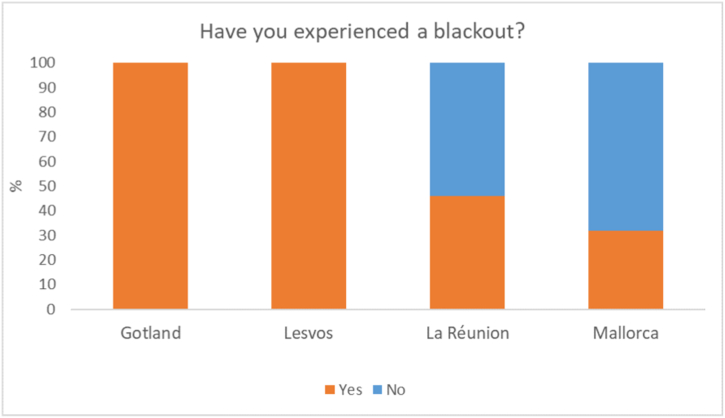
Percentage of respondents that have experienced a blackout on each of the reviewed islands.

A question was also asked if the respondent had experienced a blackout on their island. As can be seen in Fig. 4, all respondents on Gotland and Lesvos had experienced a blackout while only about half of respondents on La Réunion had. Mallorca respondents were the least likely to have experienced a blackout, with less than a third having experienced one.

“I don’t remember the last time there was a blackout.“(M04)

A follow-up question on the length of blackouts on the islands indicated that they tended to be a few hours or less on all islands. A second follow-up question asked how the respondent coped with the blackouts, to which the response was overwhelmingly to simply wait. Less than a quarter of respondents had considered finding a personal technological solution for the blackouts, though many noted they had made non-technological preparations such as having candles and flashlights on hand. Expectedly, respondents from Mallorca were the least likely to have considered a solution.

“There isn't any point in investing in a solution here, the blackouts are so short.“(G04)

“There are no blackouts, these happened before they placed the cable on the sea floor. Now the blackouts only happen when the power lines go out. A solution (for these) isn’t necessary.“(M12)

### 3.2 Respondent energy transition concept awareness

In the interviews and survey, respondents were given the opportunity to rank their self-assessed knowledge of key energy concepts related to the REACT project. These ranged from broad concepts such as saving, storing and managing energy to more specific concepts such as demand response and local energy communities. In the interviews, the respondents rated their knowledge of a given concept on a five-point Likert scale ranging from ‘Nothing’ to ‘A great deal’. The respondents were invited to enrich their answers and a specific follow-up question on where they had learned about local energy communities was also posed.

Of the broader concepts, respondents felt they had the most knowledge about saving energy while they felt they had the least about energy storage. The respondents on Gotland and Lesvos in particular had high degrees of awareness for the broader concepts with nearly all respondents on both islands having at least ‘A little bit’ of knowledge for all concepts.

**Fig. 5:**
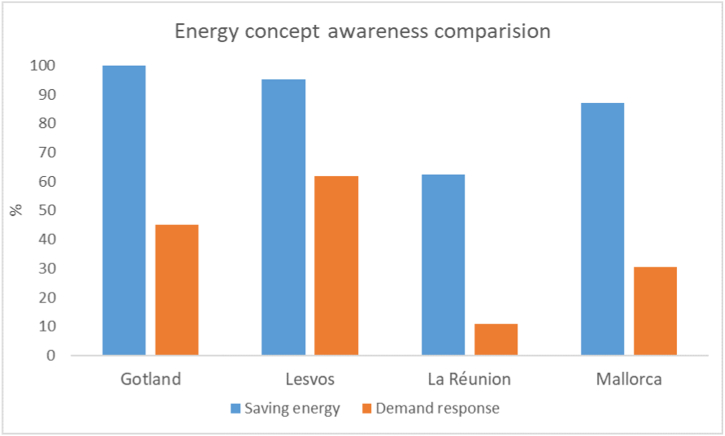
Differences in the levels of knowledge between a general concept and specific action.

For the more specific concepts, respondents on all islands expressed markedly lower levels of knowledge. For the concept of demand response, slightly more than half of the respondents from the island of Lesvos expressed at least ‘A little bit’ of knowledge of the concept. Fewer than half of the respondents from the other islands knew anything about the concept, and only a small number of La Réunion's respondents being aware of it. Respondents on the islands had even lower levels of knowledge regarding the concept of local energy communities, with the notable exception of Gotland where two-thirds had some knowledge. Fig. 5 below graphically illustrates the knowledge differences for the islands between a general concept, saving energy, and a more specific action, demand response, based on the number of respondents that stated they had at least ‘A little bit’ of knowledge on the concepts.

### 3.3 Respondent willingness to participate in energy transition activities

**Fig. 6:**
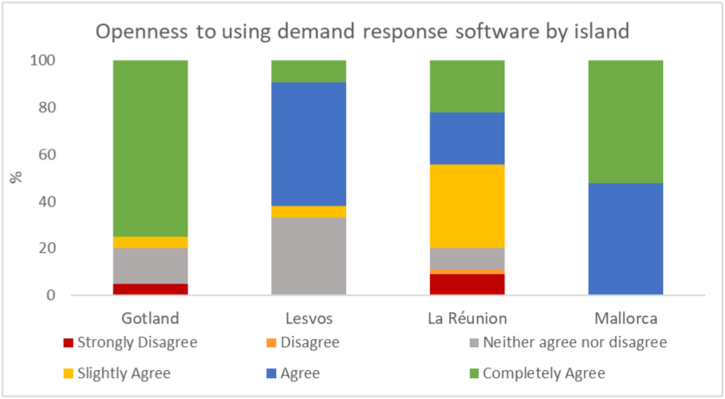
Respondent openness to using demand response software by island.

The final three questions in the interviews and survey asked about the respondent's willingness to use demand response software, their reasons for wanting or not wanting to and their concerns about using such a technology. On all islands, the respondents expressed an overall willingness to use demand response technologies, with between two-thirds on Lesvos and all respondents on Mallorca expressing at least some openness to using the technology as shown in Fig. 6 below.

**Fig. 7:**
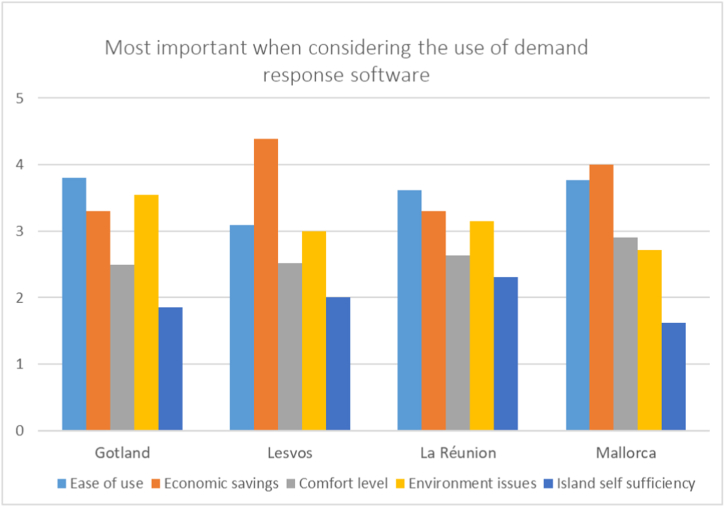
Category average importance ranking when considering the use of demand response software, by island.

The respondents were then asked to rank five attributes on how much these influenced their openness to using or not using demand response software. These attributes were Ease of use, Economic savings, Comfort level, Environmental issues and Island self-sufficiency. The Ease of use category ranked highest or next highest for all four islands while Economic savings ranked highest or next highest for all islands except Gotland. Unlike the other three islands, Gotland ranked Economic savings third and instead gave Environmental issues higher importance. Working towards island self-sufficiency was considered to be the least important reason for using demand response on all islands, though the respondents on islands with a connection to mainland grids, Gotland and Mallorca, valued it noticeably lower than those without one. Fig. 7 below details the average score for each island for each category on a 1 to 5 scale. In the Figure, higher values indicate higher stated importance.

The final question on the respondent's willingness to use a demand response tool was open-ended and gave the respondents' the opportunity to express their concerns with using a demand response solution. Most respondents across all islands didn't express a concern but for those that did, loss of control of when they could use energy for various purposes was the most common.

“I am worried that it might take a lot of time or make my life more difficult. It isn't also going to work; we live in an apartment so we can't start washing our clothes at 1 a.m. and disturb our neighbours with it.“(G06)

“It sounds interesting but it would be very difficult with kids, I think. Sometimes you really need to wash clothes and do dishes- it can't wait. I don't know if I or my family are sufficiently flexible.“(G10)

Ease of use and the data security of a demand response system were also both named as further areas of concern.

“It is important that it is easy to use because otherwise it is an unintelligible forest because customers have to learn the tariff options, and the consumer should have information about the tariffs. It should be easy for them to understand.“(M05)

“I don’t have time to play with these things, so it needs to be easy. If it isn’t user friendly, what is the point? It shouldn’t be time consuming.“(G19)

“Personal data management. This is the first important element for me. My criteria: yes if public management with full transparency and control by citizens, no management of the system by a private third party.“(R13)

Some concerns were linked to local conditions, such as some respondents’ lack of trust around the involvement of specific local authorities.

“I worry about how much control (the local grid operator) would have over when I can use things or things start.“(G02)

“Who is organizing and managing this scheme?“(L02)

### 3.4 Open-ended questions

During the interview process, the respondents were encouraged to enrich their responses to questions with their reasoning or commentary. In addition to this, a few questions sought the respondents' thoughts on energy related matters and their own understandings. One of these questions asked if there was something (energy related) they would like to learn more about and another asked the respondent to describe how they saw their island's energy management in ten years' time. Many respondents on all four islands expressed a desire to learn more about the concepts and technologies discussed in the interview, particularly if they had rated their own knowledge on one or more concepts as low. Several respondents would like more information regarding the costs and benefits of different energy technologies, most commonly solar panels and battery storage, and about the ways to find and join energy communities as well as the legal process for joining.

“How (does one) join a local energy community?“(L05)

“I would like to learn more about energy storage and get more concrete actions to change consumption habits.“(G07)

“What energy saving devices are suitable for collective housing?“(R19)

“Which actors, companies or entrepreneurs are working in Mallorca to promote distributed generation and energy communities? I would like to contact them and cooperate and work with them.“(M16)

The answers to the open question about the respondent's view on their island's future energy management were far more diffuse but were placed into three general categories – positive, neutral and negative. Answers were considered positive if the respondent used optimistic or positive language to describe their island's ability to improve its energy management overall, as exemplified by a Gotland respondent.

“I think it is going to improve a lot. There is a lot of interest in Gotland and there are a lot of pilot projects on Gotland. Things will be a lot better in 10 years.“(G11)

Neutral answers were those where the respondent did not expect significant improvements to be taken as a whole, or stated they were unsure or didn't know while also not simultaneously expressing concerns as exemplified by a Mallorcan respondent.

“I don't know, but I don't think there will be much change.“(M14)

Negative answers were those that used pessimistic language concerning expected energy management improvements and predicted or anticipated negative consequences as exemplified by a respondent from La Réunion.

“Still very dependent on fossil fuels, notably because of a strong dependence on the car, and very slow change of mentality.“(R34)

To improve the validity of the analysis, responses were independently analyzed by the authors and classified into one of the above categories [61].

**Fig. 8:**
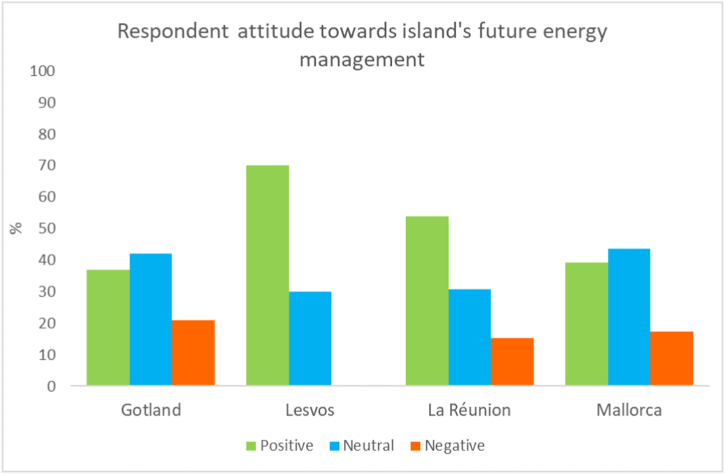
Summary attitudes on each island on the likelihood for improvement in their island's energy management in 10 years.

The positive and neutral responses regarding the respondents' assessments of their island's future energy management greatly outnumbered the negative on each island. The proportion of positive, neutral and negative answers for Gotland and Mallorca were similar while La Réunion and Lesvos' respondents were noticeably more positive, see Fig. 8.

## 4 Discussion and policy recommendations

This chapter presents the discussion on the results by category of question types, as used in Chapter 3.

### 4.1 Respondent energy literacy

The respondents’ self-reported levels of knowledge on the various energy concepts should also be taken with some degree of skepticism as research has found that self-reported levels of knowledge and actions often do not match up well with actual knowledge and actions. This discrepancy has also be found to be true for self-reported levels of energy literacy and energy actions [[63], [64], [65]]. For instance, the number of respondents that knew the cost of electricity they used each month was notably higher, especially for Lesvos, than other researchers have found among non-island dwelling residential users [44,63,66]. An increased awareness of electricity costs by island residents could potentially be the result of the generally higher costs for utility services found on islands [67,68] and on some of the islands examined in this work [69,70]. This, when combined with higher electricity prices, could result in islanders being more conscious of their electricity bills and ways to defray those costs.

The occurrence of blackouts on an island doesn't appear to influence the overall desire to take part in energy transition activities. Despite their having experienced fewer blackouts, Mallorcan respondents were generally as knowledgeable about most of the energy concepts when compared to the other islands' respondents. Further, the perceived lower incidence of blackouts on the island didn't negatively impact willingness to participate in local energy communities or use demand response software, both of which were high. At the same time, there was no significant difference when it came to respondents' desires to seek a technological solution with which they could address the blackouts between the islands with the highest perceived incidence of blackouts, Gotland and Lesvos, and the lowest, Mallorca. This lack of interest may potentially be related to the relatively short duration of the blackouts reported on all islands.

### 4.2 Concept awareness and willingness to participate in energy transition activities

There appears to be some disconnect between awareness and desire or ability to join energy communities on Gotland. Interest in joining such a community was higher on islands that had far lower levels of initial awareness of local energy communities. Two-thirds of Gotland's respondents' were initially aware of local energy communities but less than half were definitely interested in joining one. As compared to Lesvos, La Réunion and Mallorca, where initial awareness ranged from a third to less than a quarter of respondents, more than half of the respondents expressed a willingness to join. These high levels of awareness on Gotland are potentially due to the long running existence of an organization which has sought, and widely publicized its efforts, to establish a local energy community [[71], [72], [73]], a local governmental organization which specifically spreads information on local energy transition actions and the island's status as an ‘Energy pilot’ for Sweden [74]. This outreach work appears to have had an impact on Gotland's resident's awareness to the concept, despite a large number of those asked not remembering the specific source of their knowledge.

“I heard something about (local energy communities), it could have been something in the mail or maybe the local newspaper.“(G05)

“I don't know? I read about it in an article or talked with someone about it.“(G13)

Interestingly, this increased awareness has not increased their willingness to participate when compared to the other islands' respondents. This lack of willingness may stem from issues the local organization has had in establishing the envisioned community due to regulatory barriers in Sweden [75]. Similar legal barriers exist on La Réunion where the development of solutions, such as energy communities, is impeded by national law that doesn't align with the conditions on the island [21]. On the other end of the spectrum, Mallorca, with nearly half the initial awareness of Gotland, has since the interviews took place experienced changes its laws which actively support the establishment of energy communities [76,77].

This can be compared to the desire to use demand response software which was strongest on Mallorca where all respondents stated they would be open to its use and on Gotland, where three-quarters would be open to using the software, see Fig. 6. On Lesvos and La Réunion, the majority of respondents stated they would be open to using the software albeit less strongly than on the other two islands. La Réunion especially had a greater number of respondents that were only slightly in agreement with usage of the software when compared to the other islands. The respondents' reasons for considering the usage of the software aren't in close alignment for the most ‘willing’ islands. While the respondents on Gotland and Mallorca highly valued Ease of use, Gotland respondents placed a much stronger emphasis on Environmental issues than those on Mallorca. The islands with lower willingness to use a demand response solution, Lesvos and La Réunion, both emphasize Economic savings and Ease of use, though Lesvos' respondents placed significantly more emphasis on Economic savings.

Differences in energy pricing and respondent incomes across the islands could potentially explain the differences in emphasis around economic savings, though this does not seem to be the case here. Evaluation of the respondent incomes compared to their stated preferences didn't provide any clear correlation. Respondents that claimed to be in the higher income ranges (3000+ €) were nearly as likely as those claiming to be in the lower ranges to place Economic savings as their most important consideration.

That the respondents across all islands' focus on ease of use when considering whether or not to use demand response software is in line with other research on the topic [48,53,78], as was the emphasis on economic savings. Interestingly, comfort level wasn't a key consideration on any island nor was the goal of island self-sufficiency valued highly against the other criteria, which again falls in line with earlier research's motivators [49]. The demand response concerns most commonly noted by respondents in this study, namely loss of control, ease of use and data security, mirror those found in other research on demand response uptake and acceptance [49,79].

### 4.3 Open-ended questions

Another point of interest that can be taken from the open answer questions is the generally positive viewpoints respondents have for improvement in their island's energy management. This positivity, interestingly, doesn't appear to be connected to the respondents' willingness to participate in transition activities. This is exemplified by Mallorca's respondents that were the most open to using demand response software and joining an energy community but, similar to Gotland's respondents, were more reserved in their expectations for their island as a whole. Conversely, Lesvos' respondents were markedly more positive towards the development of their island's energy management but were relatively less willing to use the demand response tool or join a local energy community themselves.

### 4.4 Policy recommendations

What can be seen across all islands is that there is a distinct opportunity to engage with residents by providing information on specific actions and tools that can be used to further the island's energy transition. Closing the gap that exists between basic energy concepts and the use transition tools would allow island planners to capitalize on the interest island residents have in joining local energy communities and using demand response solutions. In the case of demand response in particular, planning strategies that increase the general awareness of it while also emphasizing its potential financial benefits and ensuring the software's ease of use, perhaps by having a local agency hosting such a software, could serve a dual function of closing gaps in awareness while also increasing willingness to join.

Deciding what to emphasize to motivate island residents to engage with demand response, energy communities or another energy transition activity will depend on the circumstances of the island. On some islands, such as the not connected to the mainland grid and savings focused Lesvos, the financial benefits of energy transition solutions should lead information efforts while on others, such as interconnected Gotland, the environmental benefits could be lifted.

Ultimately, no one strategy or solution is likely to successfully engage all residents of an island. This can be seen in the responses from several respondents across the islands in this study that expressed a lack of willingness to use a demand response solution due to their inflexible energy needs. The reasons given by these respondents for not wanting to use a demand response solution do not, however, preclude them from joining an energy community which shows the importance of developing strategies that can give a range options to residents on how they are able to participate in their island's energy transition.

The general positivity of residents to their island's energy future across all islands indicates an opening for planners and policy-makers seeking to encourage energy transition activities. This opening can, potentially, be taken advantage of by engaging their island's residents with guidelines on how, for example, energy communities could be practically established on their islands. Such locally developed guidelines could help navigate policy obstacles, like those identified in Sweden, and ultimately encourage the formation of policies centrally that encourage and ease participation. More broadly, the findings show that the best opportunities for successful future island transition activities can be reached by working together locally to spread information on these activities while working nationally to remove policy obstacles.

## 5 Conclusions

In this article, a review of more than 110 responses from residents on their awareness of basic energy concepts and understanding of the energy transition was conducted across four largely dissimilar EU islands. To this end, island residents were interviewed or surveyed regarding their understanding of their own energy usage, their awareness of basic energy concepts and tools that facilitate the energy transition, their willingness to engage in activities in line with energy transition goals and their overall opinion towards their island's energy future. Clear differences and similarities in each of these across the islands were identified for levels of understanding and awareness, desires to participate and future expectations.

The paper's three major findings were in the areas of islander awareness and engagement. The overall awareness of energy transition tools and concepts was not found to be higher on islands, highlighting areas of potential focus for island energy planners to engage with residents on. While no specific engagement methods were considered in this article, a range of activities are likely to be needed to maximize the reach of the efforts to close the awareness gap between energy concepts and transition activities. To leverage these outcomes, when national policies don't support island transition activities or are unclear, it is recommended that adapted guidance specifically directed to local needs and circumstances could serve to both inform and better direct island residents wanting to become involved. An additional finding of this work is that, once informed about local energy communities and demand response, island residents were highly interested in joining and using them. Further, the consistency of willingness to use demand response technology and join local energy communities across the islands could support that similar attitudes exist on other islands, both in and outside the EU. A final finding was a general positivity from island residents towards their islands' energy future, which corresponds to their willingness to engage in engagement transition activities.

The positions expressed by residents, analyzed together with local and national energy policies, can identify significant opportunities and potential roadblocks when planning the transition of island energy systems. While providing only a first step into how local residents’ knowledge and awareness can help guide successful island energy transition planning, this study identifies some clear knowledge gaps where island energy planners can begin engaging with residents and acting on the existing willingness to join in the transition.

## Ethics statement

This study was reviewed and approved by the University of the Aegean Committee on Research Ethics and Conduct, with the ethics approval number: 5976/22.03.2021. All participants provided informed consent to participate in the study.

## Author contribution statement

Andrew Barney: Conceived and designed the experiments; Performed the experiments; Analyzed and interpreted the data; Contributed reagents, materials, analysis tools or data; Wrote the paper. Heracles Polatidis: Conceived and designed the experiments; Analyzed and interpreted the data; Wrote the paper. Stergios Vakalis; Dominique Grondin; Fausto Sainz Salces: Performed the experiments; Analyzed and interpreted the data; Contributed reagents, materials, analysis tools or data; Wrote the paper. Michel Benne: Performed the experiments; Contributed reagents, materials, analysis tools or data; Wrote the paper. Dias Haralampopoulos: Conceived and designed the experiments; Wrote the paper.

## Data availability statement

The data that has been used is confidential.

## Declaration of competing interest

The authors declare that they have no known competing financial interests or personal relationships that could have appeared to influence the work reported in this paper.

## References

[1] Daw J., Stout S. (2019).

[2] Groppi D., Pfeifer A., Garcia D., Krajačić G., Duić N. (2021). A review on energy storage and demand side management solutions in smart energy islands. Renew. Sustain. Energy Rev..

[3] Bertheau P. (2020). Supplying not electrified islands with 100% renewable energy based micro grids: a geospatial and techno-economic analysis for the Philippines. Energy.

[4] Meschede H., Bertheau P., Khalili S., Breyer C. (2022).

[5] Marczinkowskia H.M., Østergaard P.A., Mauger R. (2022). Energy transitions on European islands: exploring technical scenarios, markets and policy proposals in Denmark, Portugal and the United Kingdom. Energy Res. Social Sci..

[6] Barney A., Polatidis H., Haralambopoulos D. (2022). Decarbonisation of islands: a multi-criteria decision analysis platform and application. Sustain. Energy Technol. Assessments.

[7] Jelić M., Batić M., Tomašević N., Barney A., Polatidis H., Crosbie T., Abi Ghanem D., Short M., Pillai G. (2020). Towards self-sustainable island grids through optimal utilization of renewable energy potential and community engagement. Energies.

[8] Barney A., Polatidis H., Jelić M., Tomašević N., Pillai G., Haralambopoulos D. (2021). Transition towards decarbonisation for islands: development of an integrated energy planning platform and application. Sustain. Energy Technol. Assessments.

[9] Barney A., Petersen U.R., Polatidis H. (2022). Energy scenarios for the Faroe Islands: a MCDA methodology including local social perspectives. Sustainable Futures.

[10] Williams S., Short M., Crosbie T., Shadman-Pajouh M. (2020). A decentralized informatics, optimization, and control framework for evolving demand response services. Energies.

[11] Abi Ghanem D., Crosbie T. (2021). The transition to clean energy: are people living in island communities ready for smart grids and demand response?. Energies.

[12] Energimyndigheten (2018).

[13] Deddie S.A., DEDDIE S.A. (2022). https://deddie.gr/el/themata-tou-diaxeiristi-mi-diasundedemenwn-nisiwn/agora-mdn/stoixeia-ekkathariseon-kai-minaion-deltion-mdn/miniaia-deltia-ape-thermikis-paragogis/minaia-pliroforiaka-deltia-paragogis-2021/.

[14] (2 December 2022). Red Eléctrica.

[15] (November 2022). Observatoire énergie réunion, "Bilan Énergétique de la Réunion.

[16] Google Earth Pro (2022).

[17] Nilsson K., Soares J., Ivanell S. (2017).

[18] Sopher D., Juhlin C., Levendal T., Erlström M., Nilsson K., Soares J. (2019).

[19] Wallnerstörm C., Bertling Tjernberg L. (2018).

[20] Praene J.P., David M., Sinama F., Morau D., Marc O. (2012). Renewable energy: progressing towards a net zero energy island, the case of Reunion Island. Renew. Sustain. Energy Rev..

[21] Sawatzky M., Albrecht M. (2017). Translating EU renewable energy policy for insular energy systems: reunion Island's quest for energy autonomy. Fennia - International Journal of Geography.

[22] Bénard-Sora F., Praene J.P. (2018). Sustainable urban planning for a successful energy transition on Reunion Island: from policy intentions to practical achievement. Util. Pol..

[23] Selosse S., Garabedian S., Ricci O., Maïzi N. (2018). The renewable energy revolution of reunion island. Renew. Sustain. Energy Rev..

[24] Babonneau F., Biscaglia S., Chotard D., Haurie A., Mairet N., Lefillatre T. (2021). Assessing a transition to 100% renewable power generation in a non-interconnected area: a case study for La réunion island. Environ. Model. Assess..

[25] Razumova M., Ibáñez J.L., Palmer J.R.M. (2015). Drivers of environmental innovation in Majorcan hotels. J. Sustain. Tourism.

[26] Blázquez-Salom M., Cladera M., Sard M. (2021). Identifying the sustainability indicators of overtourism and undertourism in Majorca. J. Sustain. Tourism.

[27] García-Buades M.E., García-Sastre M.A., Alemany-Hormaeche M. (2022). Effects of overtourism, local government, and tourist behavior on residents' perceptions in Alcúdia (Majorca, Spain). Journal of Outdoor Recreation and Tourism.

[28] Curto D., Franzitta V., Viola A., Cirrincione M., Mohammadi A., Kumar A. (2019). A renewable energy mix to supply small islands. A comparative study applied to Balearic Islands and Fiji. J. Clean. Prod..

[29] Torres C., Jordà G., de Vílchez P., Vaquer-Sunyer R., Rita J., Canals V., Cladera A., Escalona J., Miranda M. (2021). Climate change and its impacts in the Balearic Islands: a guide for policy design in Mediterranean regions. Reg. Environ. Change.

[30] Tegou L.-I., Polatidis H., Haralambopoulos D.A. (2012). A multi-criteria framework for an isolated electricity system design with renewable energy sources in the context of distributed generation: the case study of Lesvos island, Greece. Int. J. Green Energy.

[31] Kapsali M., Anagnostopoulos J., Kaldellis J. (2012). Wind powered pumped-hydro storage systems for remote islands: a complete sensitivity analysis based on economic perspectives. Appl. Energy.

[32] Psarros G.N., Nanou S.I., Papaefthymiou S.V., Papathanassiou S.A. (2018). Generation scheduling in non-interconnected islands with high RES penetration. Renew. Energy.

[33] Kapsali M., Anagnostopoulos J. (2017). Investigating the role of local pumped-hydro energy storage in interconnected island grids with high wind power generation. Renew. Energy.

[34] Sovacool B. (2014). What are we doing here? Analyzing fifteen years of energy scholarship and proposing a social science research agenda. Energy Res. Social Sci..

[35] Tampakis S., Τsantopoulos G., Arabatzis G., Rerras I. (2013). Citizens' views on various forms of energy and their contribution to the environment. Renew. Sustain. Energy Rev..

[36] Wüstenhagen R., Wolsink M., Bürer M.J. (2007). Social acceptance of renewable energy innovation: an introduction to the concept. Energy Pol..

[37] von Wirth T., Gislason L., Seidl R. (2018). Distributed energy systems on a neighborhood scale: reviewing drivers of and barriers to social acceptance. Renew. Sustain. Energy Rev..

[38] Stephanides P., Chalvatzis K., Li X., Lettice F., Guan D., Ioannidis A., Zafirakis D., Papapostolou C. (2019). The social perspective on island energy transitions: evidence from the Aegean archipelago. Appl. Energy.

[39] Demetriou D., Polatidis H., Haralambopoulos D. (2014). Integrated energy planning for the residential sector: the case study of Cyprus. Energy Sources B Energy Econ. Plann..

[40] Pacheco A., Monteiro J., Santos J., Sequeira C., Nunes J. (2022). Energy transition process and community engagement on geographic islands: the case of Culatra Island (Ria Formosa, Portugal). Renew. Energy.

[41] Heaslip E., Fahy F. (2018). Developing transdisciplinary approaches to community energy transitions: an island case study. Energy Res. Social Sci..

[42] Skiniti G., Daras T., Tsoutsos T. (2022). Analysis of the community acceptance factors for potential wind energy projects in Greece. Sustainability.

[43] van den Broek K.L. (2019). Household energy literacy: a critical review and a conceptual typology. Energy Res. Social Sci..

[44] Brounen D., Kok N., Quigley J. (2013). Energy literacy, awareness, and conservation behavior of residential households. Energy Econ..

[45] Biresselioglu M., Demir M., Kaplan M., Solak B. (2020). Individuals, collectives, and energy transition: analysing the motivators and barriers of European decarbonisation. Energy Res. Social Sci..

[46] Liu W., Wang C., Mol A.P. (2013). Rural public acceptance of renewable energy deployment: the case of Shandong in China. Appl. Energy.

[47] Bugden D., Stedman R. (2019). A synthetic view of acceptance and engagement with smart meters in the United States. Energy Res. Social Sci..

[48] Kardooni R., Yusoff S.B., Kari F.B. (2016). Renewable energy technology acceptance in Peninsular Malaysia. Energy Pol..

[49] Parrish B., Heptonstall P., Gross R., Sovacool B. (2020). A systematic review of motivations, enablers and barriers for consumer engagement with residential demand response. Energy Pol..

[50] Davis F. (1989). Perceived usefulness, perceived ease of use, and user acceptance of information technology. MIS Q..

[51] Tsaur R., Lin Y. (2018). Exploring the consumer attitude of building-attached photovoltaic equipment using revised technology acceptance model. Sustainability.

[52] Chen C.F., Xu X., Arpan L. (2017). Between the technology acceptance model and sustainable energy technology acceptance model: investigating smart meter acceptance in the United States. Energy Res. Social Sci..

[53] Broman Toft M., Schuitema G., Thøgersen J. (2014). Responsible technology acceptance: model development and application to consumer acceptance of Smart Grid technology. Appl. Energy.

[54] Wang N., Hangqi T., Zhu S., Li Y. (2022). Analysis of public acceptance of electric vehicle charging scheduling based on the technology acceptance model. Energy.

[55] Siano P. (2014). Demand response and smart grids—a survey. Renewable Sustainable Energy Rev..

[56] O׳Connell N., Pinson P., Madsen H., O׳Malley M. (2014). Benefits and challenges of electrical demand response: a critical review. Renewable Sustainable Energy Rev..

[57] Guest G., Bunce A., Johnson L. (2006). How many interviews are enough?: an experiment with data saturation and variability. Field Methods.

[58] Robinson O. (2013). Sampling in interview-based qualitative research: a theoretical and practical guide. Qual. Res. Psychol..

[59] Noy C. (2008). Sampling knowledge: the hermeneutics of snowball sampling in qualitative research. Int. J. Soc. Res. Methodol..

[60] Molin E., Steg L. (2012). Psychological factors influencing sustainable energy technology acceptance: a review-based comprehensive framework. Renew. Sustain. Energy Rev..

[61] Bengtsson M. (2016). How to plan and perform a qualitative study using content analysis. NursingPlus Open.

[62] Carley K., Asher R. (1993). The Encyclopedia of Language and Linguistics.

[63] Sovacool B., Blyth P. (2015). Energy and environmental attitudes in the green state of Denmark: implications for energy democracy, low carbon transitions, and energy literacy. Environ. Sci. Pol..

[64] Murtagh N., Nati M., Headley W.R., Gatersleben B., Gluhak A., Imran M.A., Uzzell D. (2013). Individual energy use and feedback in an office setting: a field trial. Energy Pol..

[65] Murphy T. (2002).

[66] Boogen N., Filippini M., Kumar N., Blasch J. (2018).

[67] Notton G. (2015). Importance of islands in renewable energy production and storage: the situation of the French islands. Renewable Sustainable Energy Rev..

[68] Armstrong H.W., Read R.C. (2021). The non-sovereign territories: economic and environmental challenges of sectoral and geographic over-specialisation in tourism and financial services. Eur. Urban Reg. Stud..

[69] Govern de les Illes Balears (2015).

[70] Fintikakis G. (2020). https://www.e-mc2.gr/en/node/7698.

[71] energi Austerland (2022). https://austerlandenergi.se/.

[72] Linusson L. (February 2018). https://helagotland.se/samhalle/austerland-energi-vill-se-ett-fossilfritt-samhalle-15077639.aspx.

[73] Neuman J. (2020).

[74] Gotland Region (July 2022). https://www.gotland.se/energipilot.

[75] Palm J., Boije E., af Gennäs Erre (2022).

[76] Oficial del Estado Boletín (2022).

[77] Renovables Energías (September 2022). https://www.energias-renovables.com/autoconsumo/baleares-abre-la-primera-linea-de-ayudas-20220927.

[78] Naghiyev E., Shipman R., Goulden M., Gillott M., Spence A. (2022). Cost, context, or convenience? Exploring the social acceptance of demand response in the United Kingdom. Energy Res. Social Sci..

[79] Murtagh N., Gatersleben B., Uzzell D. (2014). A qualitative study of perspectives on household and societal impacts of demand response. Technol. Anal. Strat. Manag..

