# Supplementary material for: Energy transition awareness: Can it guide local transition planning on islands?
Source: Heliyon. 2023 Sep 9;9(9):e19960. doi: 10.1016/j.heliyon.2023.e19960 (PMC10559555; doi:10.1016/j.heliyon.2023.e19960)
Supplement: Multimedia component 1 [file mmc1.docx]

# Appendix A: Interview question template

**Introduction**

- Welcome
- Thank the person for answering the call, attending the appointment, etc.
- Introduce yourself and the project

**H2020 REACT**

**Welcome to the project’s survey!**

Thank you for taking part in our survey. The goal behind this survey is be get a better understanding for your energy consumption and the energy consuming and producing equipment in your home. This survey is a part of the research project REACT that is financed by the European Commission. The goal of the REACT project is to reduce energy costs for island energy communities and to develop renewable energy solutions on [Island name] to improve local energy services and the environment.

To take part, please read the following and check the box:

🞏 I would like to take part in the interview. I understand that it is confidential, that I will not be identified and that my answers will be anonymized if nothing else has been specifically stated. I understand that my information will only be accessible to the project group and the project partner’s organizations. My information will be stored in a secure manner and will be destroyed at the project’s conclusion in accordance with EU directive.

## Definitions of the main terms

**Demand response** – changes in power consumption by an electricity customer to match more closely their demand for electricity to its supply.

**Saving energy** – changes in power consumption by a customer of electricity to reduce their demand.

**Energy management** – the process of tracking and optimizing energy consumption to conserve its usage.

**Local energy communities** – open and voluntary collective energy actions by citizens, local authorities and small businesses who are not involved in the energy sector which seek to generate social and environmental benefits rather than financial profits.

**Energy storage** – a means of saving generated energy for later use, for instance in batteries, heat tanks or pumped hydro solutions.

## Respondent Characteristics

| **Gender** |  |
| --- | --- |
| Female |  |
| Male |  |
| Other |  |
| Do not wish to answer |  |
| **Age** |  |
| 18–24 years |  |
| 25–29 years |  |
| 30–39 years |  |
| 40–49 years |  |
| 50–64 years |  |
| 65 years and older |  |
| **Highest professional qualiﬁcation** |  |
| No professional qualiﬁcation |  |
| Apprenticeship, vocational training |  |
| Certiﬁcate from a technical college |  |
| Qualiﬁcation from a specialised academy or a college of advanced vocational studies |  |
| Qualiﬁcation from a university of applied sciences |  |
| University degree |  |
| Doctorate |  |
| **Employment status** |  |
| Employed |  |
| Unemployed |  |
| Pensioner |  |
| Pupil/student |  |
| Other |  |
| **Average monthly net household income (in EURO)** |  |
| Less than 1,000 |  |
| 1000–2000 |  |
| 2001–3000 |  |
| 3001–4000 |  |
| 4001–5000 |  |
| More than 5000 |  |
| Do not know/Do not wish to answer |  |
| **Type of community** |  |
| Urban |  |
| Suburban |  |
| Rural |  |

# Questions

1. Are you aware of your/your business’s energy usage? Has it gone up or down over time?
2. What do you think are the big energy consuming devices at your home/business?
3. Do you have any energy producing equipment, like solar panels, installed?
   1. If so, do you use net-metering?
4. Do you own an electrical car, cart or bike?
   1. If not, are you planning on purchasing one?
5. Are you aware that electricity can have a different price depending on when it is used and, if so, do you consider this when you use electricity?
6. Have you experienced a blackout? How do you handle the blackouts on the island? Have you thought of finding a solution of your own?
7. How much do you know about demand response?
   (Interviewer: Please write down response if possible and adapt answer to the following scale)

| Nothing |  | A little bit |  | Something |  | A fair amount |  | A great deal |  |
| --- | --- | --- | --- | --- | --- | --- | --- | --- | --- |

1. How much do you know about saving energy?
   (Interviewer: Please write down response if possible and adapt answer to the following scale)

| Nothing |  | A little bit |  | Something |  | A fair amount |  | A great deal |  |
| --- | --- | --- | --- | --- | --- | --- | --- | --- | --- |

1. How much do you know about energy management?
   (Interviewer: Please write down response if possible and adapt answer to the following scale)

| Nothing |  | A little bit |  | Something |  | A fair amount |  | A great deal |  |
| --- | --- | --- | --- | --- | --- | --- | --- | --- | --- |

1. How much do you know about local energy communities?
   (Interviewer: Please write down response if possible and adapt answer to the following scale)

| Nothing |  | A little bit |  | Something |  | A fair amount |  | A great deal |  |
| --- | --- | --- | --- | --- | --- | --- | --- | --- | --- |

1. How much do you know about energy storage?
   (Interviewer: Please write down response if possible and adapt answer to the following scale)

| Nothing |  | A little bit |  | Something |  | A fair amount |  | A great deal |  |
| --- | --- | --- | --- | --- | --- | --- | --- | --- | --- |

1. How did you first hear about local energy communities?
2. Would you be interested in joining a local energy community?
3. Is there something else you would like to learn more about? If so, what?
4. How do you see energy management on your island in 10 years?
5. (Additional questions or comments that came up?)

**Technology Acceptance Model (TAM) questions**

1. I would be open to using demand response software that could help me control my energy consumption

| **1** | **2** | **3** | **4** | **5** | **6** | **7** |
| --- | --- | --- | --- | --- | --- | --- |
| Strongly disagree | Disagree | Slightly disagree | Neither agree nor disagree | Slightly agree | Agree | Strongly agree |

1. Rank the following in order of importance for you when considering using demand response methods:

(1 most important, 5 least)

| Ease of use | Economic savings | Comfort level | Environment issues | Island self sufficiency |
| --- | --- | --- | --- | --- |

1. Do you have concerns about using a demand response solution? If so, what are they?
